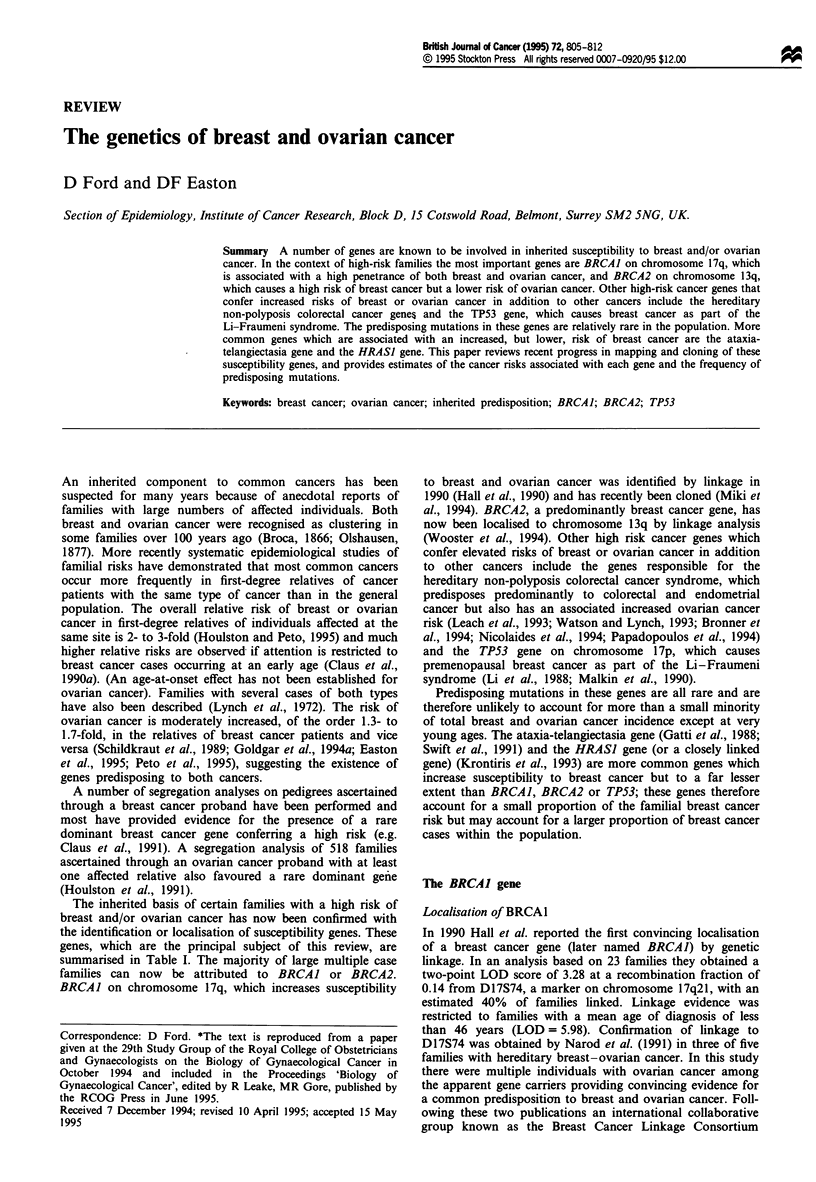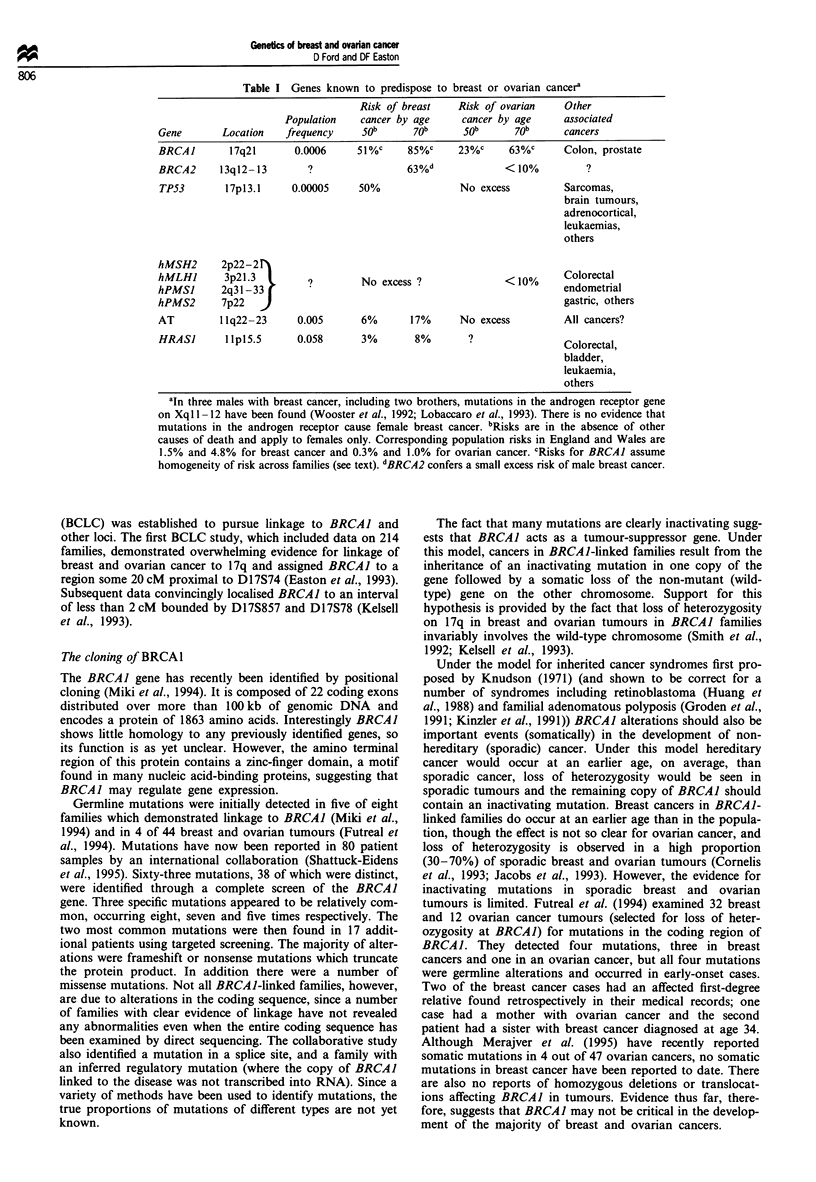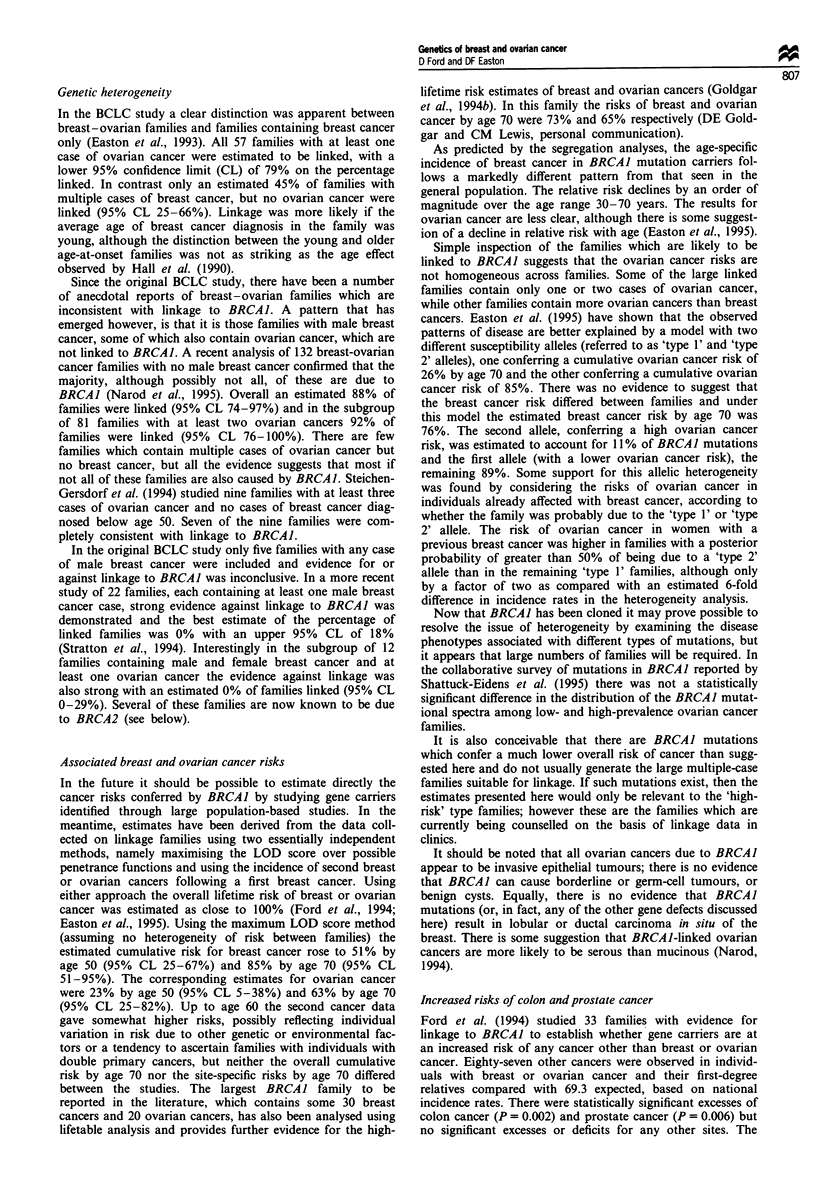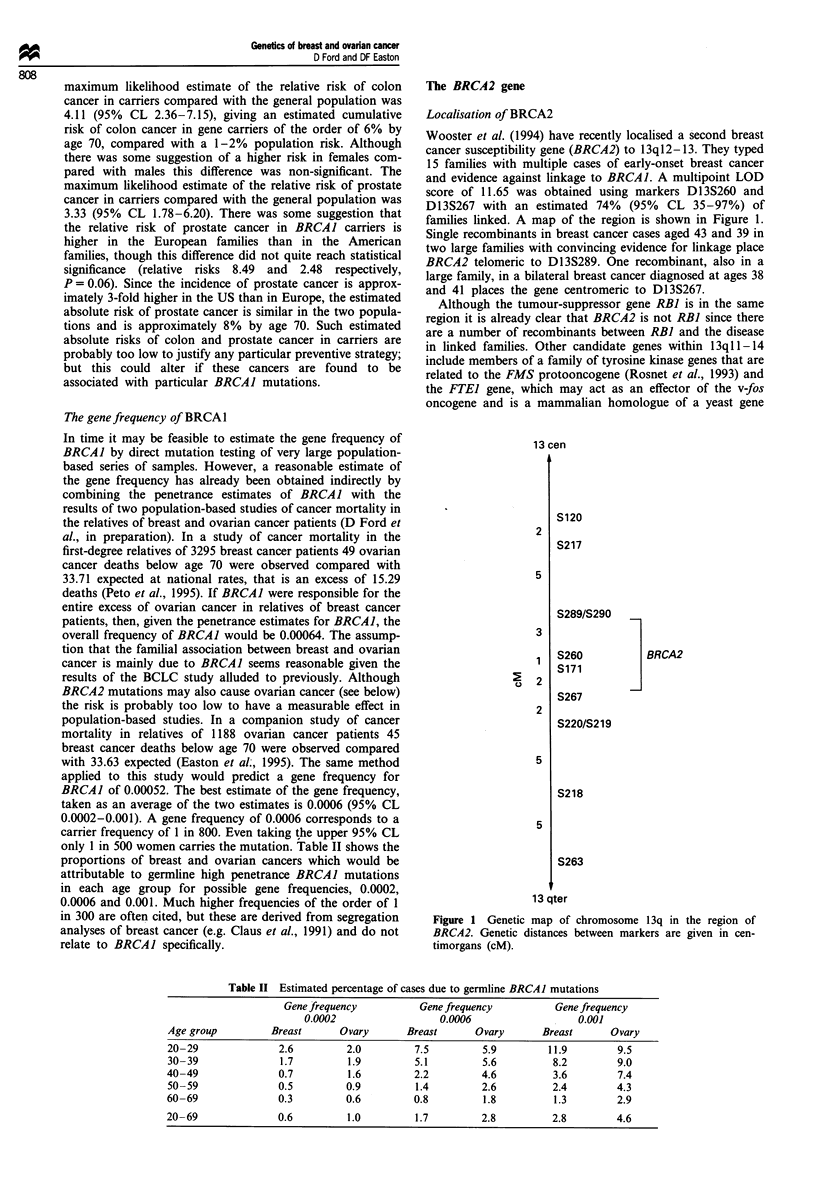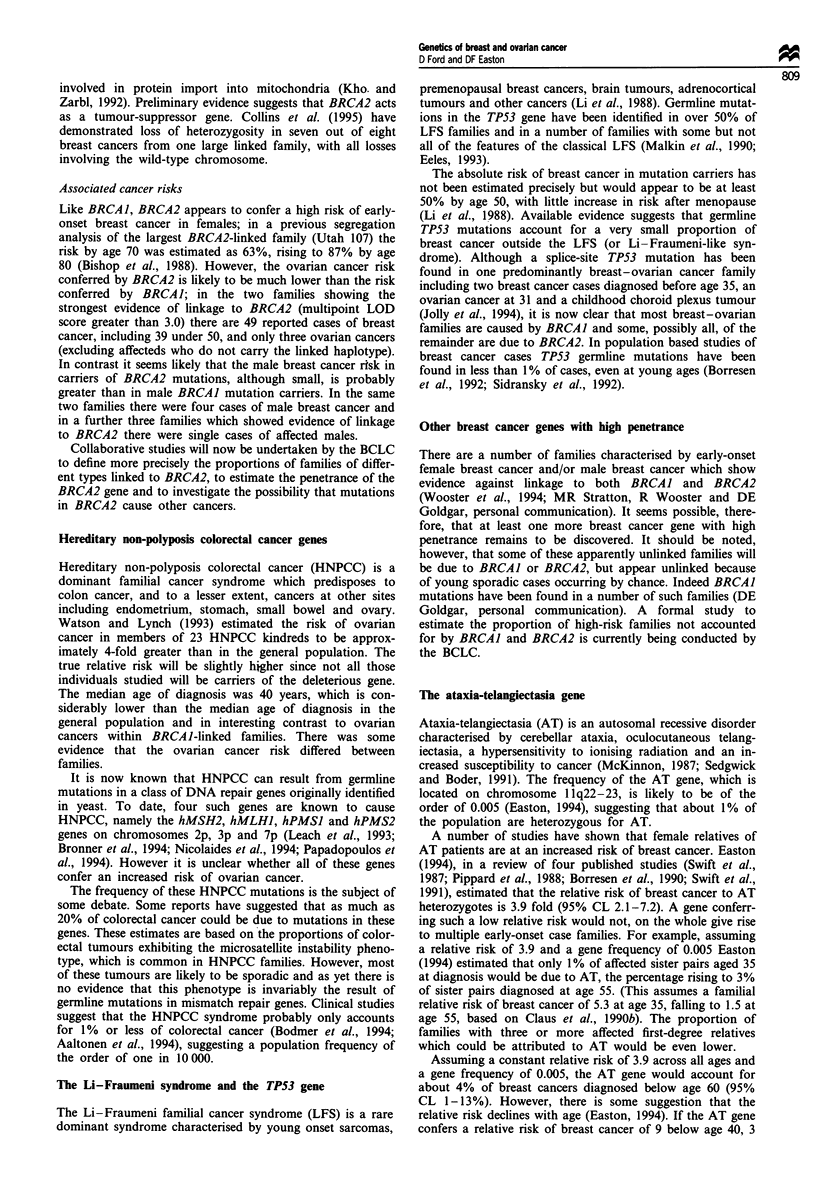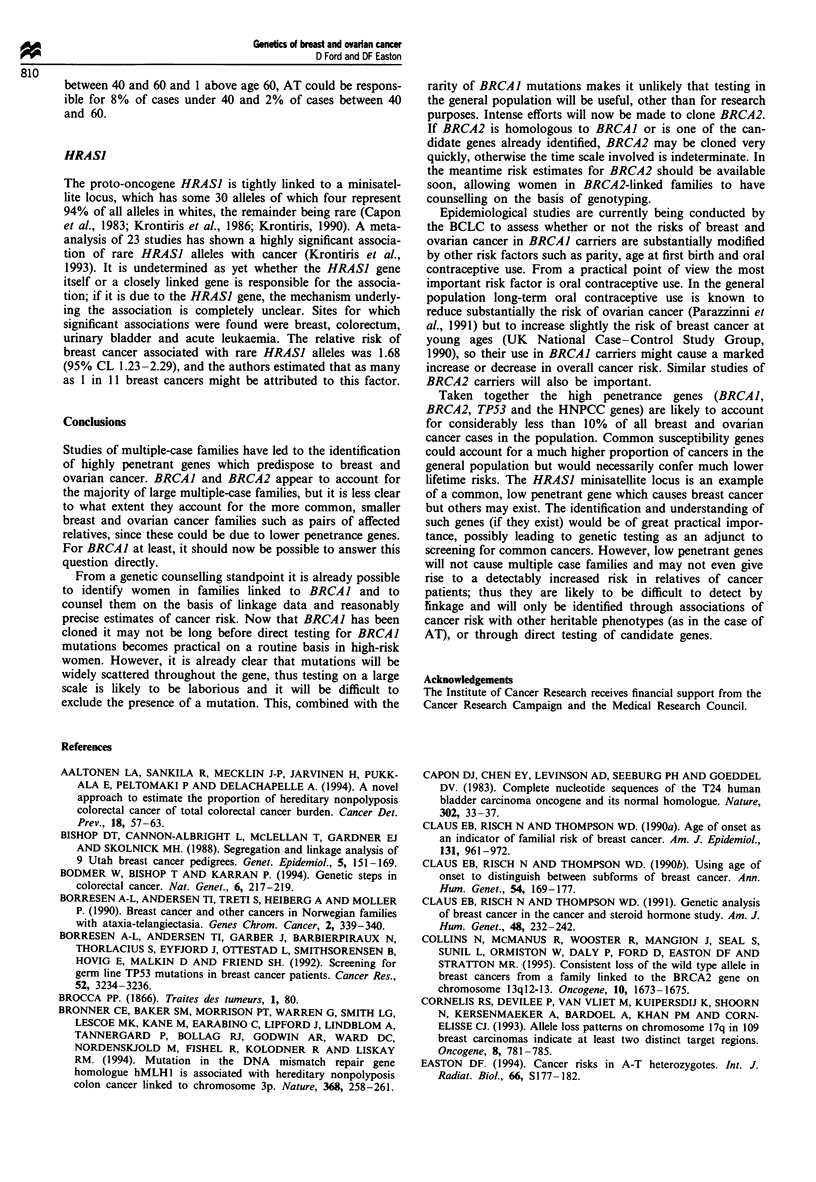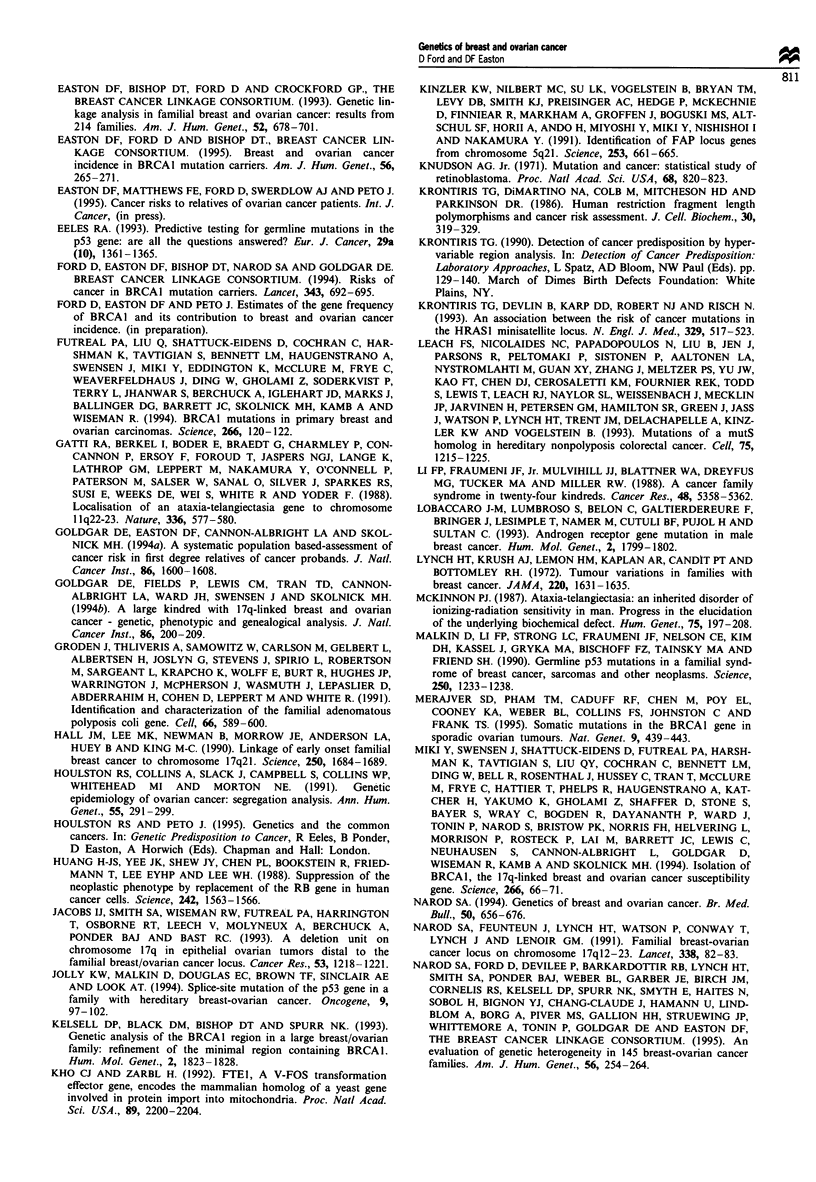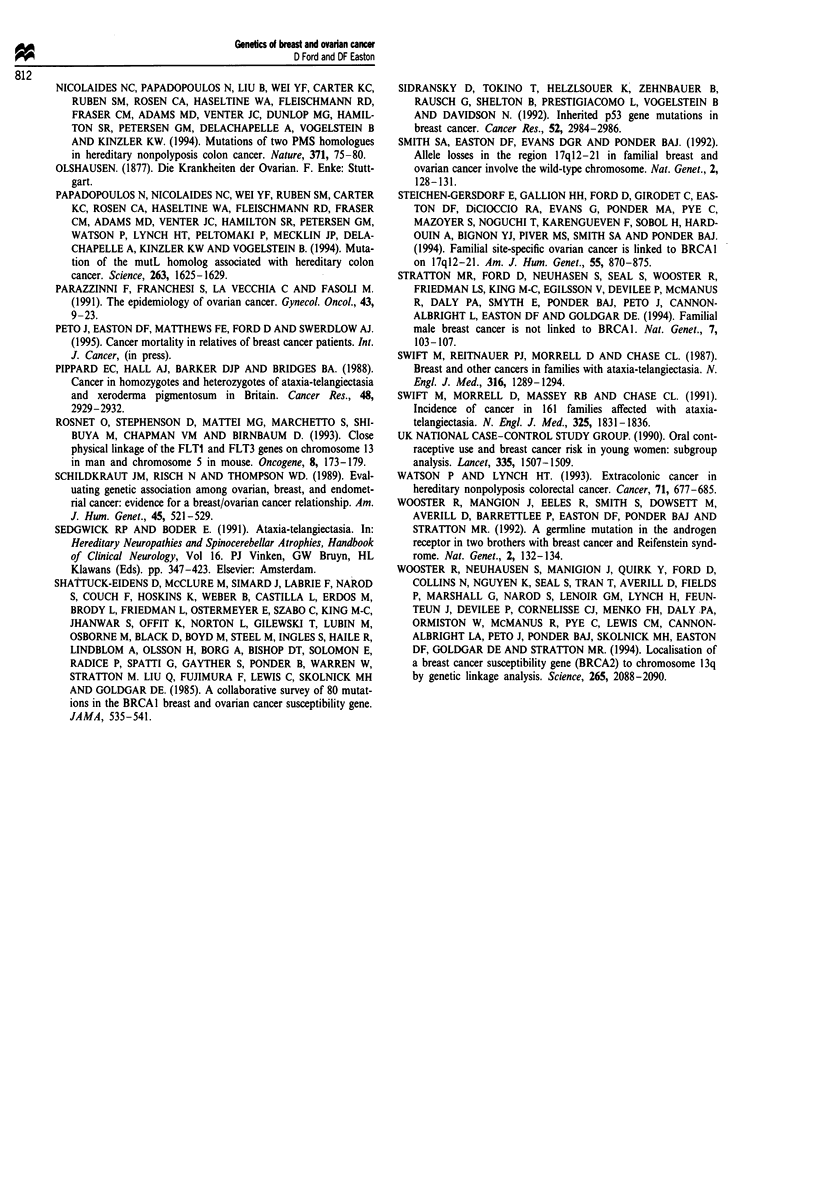# The genetics of breast and ovarian cancer.

**DOI:** 10.1038/bjc.1995.417

**Published:** 1995-10

**Authors:** D. Ford, D. F. Easton

**Affiliations:** Section of Epidemiology, Institute of Cancer Research, Belmont, Surrey, UK.

## Abstract

A number of genes are known to be involved in inherited susceptibility to breast and/or ovarian cancer. In the context of high-risk families the most important genes are BRCA1 on chromosome 17q, which is associated with a high penetrance of both breast and ovarian cancer, and BRCA2 on chromosome 13q, which causes a high risk of breast cancer but a lower risk of ovarian cancer. Other high-risk cancer genes that confer increased risks of breast or ovarian cancer in addition to other cancers include the hereditary non-polyposis colorectal cancer genes and the TP53 gene, which causes breast cancer as part of the Li-Fraumeni syndrome. The predisposing mutations in these genes are relatively rare in the population. More common genes which are associated with an increased, but lower, risk of breast cancer are the ataxiatelangiectasia gene and the HRAS1 gene. This paper reviews recent progress in mapping and cloning of these susceptibility genes, and provides estimates of the cancer risks associated with each gene and the frequency of predisposing mutations.


British Journal of Cancer (1995) 72, 805-812
© 1995 Stockton Press All rights reserved 0007-0920/95 $12.00

REVIEW

### The genetics of breast and ovarian cancer

D Ford and DF Easton

*Section of Epidemiology, Institute of Cancer Research, Block D, 15 Cotswold Road, Belmont, Surrey SM2 5NG, UK.*

**Summary** A number of genes are known to be involved in inherited susceptibility to breast and/or ovarian cancer. In the context of high-risk families the most important genes are *BRCA1* on chromosome 17q, which is associated with a high penetrance of both breast and ovarian cancer, and *BRCA2* on chromosome 13q, which causes a high risk of breast cancer but a lower risk of ovarian cancer. Other high-risk cancer genes that confer increased risks of breast or ovarian cancer in addition to other cancers include the hereditary non-polyposis colorectal cancer genes and the TP53 gene, which causes breast cancer as part of the Li-Fraumeni syndrome. The predisposing mutations in these genes are relatively rare in the population. More common genes which are associated with an increased, but lower, risk of breast cancer are the ataxia-telangiectasia gene and the *HRAS1* gene. This paper reviews recent progress in mapping and cloning of these susceptibility genes, and provides estimates of the cancer risks associated with each gene and the frequency of predisposing mutations.

**Keywords:** breast cancer; ovarian cancer; inherited predisposition; *BRCA1*; *BRCA2*; *TP53*

An inherited component to common cancers has been suspected for many years because of anecdotal reports of families with large numbers of affected individuals. Both breast and ovarian cancer were recognised as clustering in some families over 100 years ago (Broca, 1866; Olshausen, 1877). More recently systematic epidemiological studies of familial risks have demonstrated that most common cancers occur more frequently in first-degree relatives of cancer patients with the same type of cancer than in the general population. The overall relative risk of breast or ovarian cancer in first-degree relatives of individuals affected at the same site is 2- to 3-fold (Houlston and Peto, 1995) and much higher relative risks are observed if attention is restricted to breast cancer cases occurring at an early age (Claus *et al.*, 1990*a*). (An age-at-onset effect has not been established for ovarian cancer). Families with several cases of both types have also been described (Lynch *et al.*, 1972). The risk of ovarian cancer is moderately increased, of the order 1.3- to 1.7-fold, in the relatives of breast cancer patients and vice versa (Schildkraut *et al.*, 1989; Goldgar *et al.*, 1994*a*; Easton *et al.*, 1995; Peto *et al.*, 1995), suggesting the existence of genes predisposing to both cancers.

A number of segregation analyses on pedigrees ascertained through a breast cancer proband have been performed and most have provided evidence for the presence of a rare dominant breast cancer gene conferring a high risk (e.g. Claus *et al.*, 1991). A segregation analysis of 518 families ascertained through an ovarian cancer proband with at least one affected relative also favoured a rare dominant gene (Houlston *et al.*, 1991).

The inherited basis of certain families with a high risk of breast and/or ovarian cancer has now been confirmed with the identification or localisation of susceptibility genes. These genes, which are the principal subject of this review, are summarised in Table I. The majority of large multiple case families can now be attributed to *BRCA1* or *BRCA2*. *BRCA1* on chromosome 17q, which increases susceptibility to breast and ovarian cancer was identified by linkage in 1990 (Hall *et al.*, 1990) and has recently been cloned (Miki *et al.*, 1994). *BRCA2*, a predominantly breast cancer gene, has now been localised to chromosome 13q by linkage analysis (Wooster *et al.*, 1994). Other high risk cancer genes which confer elevated risks of breast or ovarian cancer in addition to other cancers include the genes responsible for the hereditary non-polyposis colorectal cancer syndrome, which predisposes predominantly to colorectal and endometrial cancer but also has an associated increased ovarian cancer risk (Leach *et al.*, 1993; Watson and Lynch, 1993; Bronner *et al.*, 1994; Nicolaides *et al.*, 1994; Papadopoulos *et al.*, 1994) and the *TP53* gene on chromosome 17p, which causes premenopausal breast cancer as part of the Li-Fraumeni syndrome (Li *et al.*, 1988; Malkin *et al.*, 1990).

Predisposing mutations in these genes are all rare and are therefore unlikely to account for more than a small minority of total breast and ovarian cancer incidence except at very young ages. The ataxia-telangiectasia gene (Gatti *et al.*, 1988; Swift *et al.*, 1991) and the *HRAS1* gene (or a closely linked gene) (Krontiris *et al.*, 1993) are more common genes which increase susceptibility to breast cancer but to a far lesser extent than *BRCA1*, *BRCA2* or *TP53*; these genes therefore account for a small proportion of the familial breast cancer risk but may account for a larger proportion of breast cancer cases within the population.

### The *BRCA1* gene

### *Localisation of* BRCA1

In 1990 Hall *et al.* reported the first convincing localisation of a breast cancer gene (later named *BRCA1*) by genetic linkage. In an analysis based on 23 families they obtained a two-point LOD score of 3.28 at a recombination fraction of 0.14 from D17S74, a marker on chromosome 17q21, with an estimated 40% of families linked. Linkage evidence was restricted to families with a mean age of diagnosis of less than 46 years (LOD = 5.98). Confirmation of linkage to D17S74 was obtained by Narod *et al.* (1991) in three of five families with hereditary breast-ovarian cancer. In this study there were multiple individuals with ovarian cancer among the apparent gene carriers providing convincing evidence for a common predisposition to breast and ovarian cancer. Following these two publications an international collaborative group known as the Breast Cancer Linkage Consortium

Correspondence: D Ford. *The text is reproduced from a paper given at the 29th Study Group of the Royal College of Obstetricians and Gynaecologists on the Biology of Gynaecological Cancer in October 1994 and included in the Proceedings 'Biology of Gynaecological Cancer', edited by R Leake, MR Gore, published by the RCOG Press in June 1995.

Received 7 December 1994; revised 10 April 1995; accepted 15 May 1995

**Table I** Genes known to predispose to breast or ovarian cancer[a]

| Gene | Location | Population frequency | Risk of breast cancer by age 50[b] | Risk of breast cancer by age 70[b] | Risk of ovarian cancer by age 50[b] | Risk of ovarian cancer by age 70[b] | Other associated cancers |
|---|---|---|---|---|---|---|---|
| *BRCA1* | 17q21 | 0.0006 | 51%[c] | 85%[c] | 23%[c] | 63%[c] | Colon, prostate |
| *BRCA2* | 13q12–13 | ? | | 63%[d] | | <10% | ? |
| *TP53* | 17p13.1 | 0.00005 | 50% | | No excess | | Sarcomas, brain tumours, adrenocortical, leukaemias, others |
| *hMSH2* <br> *hMLH1* <br> *hPMS1* <br> *hPMS2* | 2p22–21 <br> 3p21.3 <br> 2q31–33 <br> 7p22 | ? | No excess ? | | | <10% | Colorectal endometrial gastric, others |
| AT | 11q22–23 | 0.005 | 6% | 17% | No excess | | All cancers? |
| *HRAS1* | 11p15.5 | 0.058 | 3% | 8% | ? | | Colorectal, bladder, leukaemia, others |

[a]In three males with breast cancer, including two brothers, mutations in the androgen receptor gene on Xq11–12 have been found (Wooster *et al.*, 1992; Lobaccaro *et al.*, 1993). There is no evidence that mutations in the androgen receptor cause female breast cancer. [b]Risks are in the absence of other causes of death and apply to females only. Corresponding population risks in England and Wales are 1.5% and 4.8% for breast cancer and 0.3% and 1.0% for ovarian cancer. [c]Risks for *BRCA1* assume homogeneity of risk across families (see text). [d]*BRCA2* confers a small excess risk of male breast cancer.

(BCLC) was established to pursue linkage to *BRCA1* and other loci. The first BCLC study, which included data on 214 families, demonstrated overwhelming evidence for linkage of breast and ovarian cancer to 17q and assigned *BRCA1* to a region some 20 cM proximal to D17S74 (Easton *et al.*, 1993). Subsequent data convincingly localised *BRCA1* to an interval of less than 2 cM bounded by D17S857 and D17S78 (Kelsell *et al.*, 1993).

### *The cloning of* BRCA1

The *BRCA1* gene has recently been identified by positional cloning (Miki *et al.*, 1994). It is composed of 22 coding exons distributed over more than 100 kb of genomic DNA and encodes a protein of 1863 amino acids. Interestingly *BRCA1* shows little homology to any previously identified genes, so its function is as yet unclear. However, the amino terminal region of this protein contains a zinc-finger domain, a motif found in many nucleic acid-binding proteins, suggesting that *BRCA1* may regulate gene expression.

Germline mutations were initially detected in five of eight families which demonstrated linkage to *BRCA1* (Miki *et al.*, 1994) and in 4 of 44 breast and ovarian tumours (Futreal *et al.*, 1994). Mutations have now been reported in 80 patient samples by an international collaboration (Shattuck-Eidens *et al.*, 1995). Sixty-three mutations, 38 of which were distinct, were identified through a complete screen of the *BRCA1* gene. Three specific mutations appeared to be relatively common, occurring eight, seven and five times respectively. The two most common mutations were then found in 17 additional patients using targeted screening. The majority of alterations were frameshift or nonsense mutations which truncate the protein product. In addition there were a number of missense mutations. Not all *BRCA1*-linked families, however, are due to alterations in the coding sequence, since a number of families with clear evidence of linkage have not revealed any abnormalities even when the entire coding sequence has been examined by direct sequencing. The collaborative study also identified a mutation in a splice site, and a family with an inferred regulatory mutation (where the copy of *BRCA1* linked to the disease was not transcribed into RNA). Since a variety of methods have been used to identify mutations, the true proportions of mutations of different types are not yet known.

The fact that many mutations are clearly inactivating suggests that *BRCA1* acts as a tumour-suppressor gene. Under this model, cancers in *BRCA1*-linked families result from the inheritance of an inactivating mutation in one copy of the gene followed by a somatic loss of the non-mutant (wild-type) gene on the other chromosome. Support for this hypothesis is provided by the fact that loss of heterozygosity on 17q in breast and ovarian tumours in *BRCA1* families invariably involves the wild-type chromosome (Smith *et al.*, 1992; Kelsell *et al.*, 1993).

Under the model for inherited cancer syndromes first proposed by Knudson (1971) (and shown to be correct for a number of syndromes including retinoblastoma (Huang *et al.*, 1988) and familial adenomatous polyposis (Groden *et al.*, 1991; Kinzler *et al.*, 1991)) *BRCA1* alterations should also be important events (somatically) in the development of non-hereditary (sporadic) cancer. Under this model hereditary cancer would occur at an earlier age, on average, than sporadic cancer, loss of heterozygosity would be seen in sporadic tumours and the remaining copy of *BRCA1* should contain an inactivating mutation. Breast cancers in *BRCA1*-linked families do occur at an earlier age than in the population, though the effect is not so clear for ovarian cancer, and loss of heterozygosity is observed in a high proportion (30–70%) of sporadic breast and ovarian tumours (Cornelis *et al.*, 1993; Jacobs *et al.*, 1993). However, the evidence for inactivating mutations in sporadic breast and ovarian tumours is limited. Futreal *et al.* (1994) examined 32 breast and 12 ovarian cancer tumours (selected for loss of heterozygosity at *BRCA1*) for mutations in the coding region of *BRCA1*. They detected four mutations, three in breast cancers and one in an ovarian cancer, but all four mutations were germline alterations and occurred in early-onset cases. Two of the breast cancer cases had an affected first-degree relative found retrospectively in their medical records; one case had a mother with ovarian cancer and the second patient had a sister with breast cancer diagnosed at age 34. Although Merajver *et al.* (1995) have recently reported somatic mutations in 4 out of 47 ovarian cancers, no somatic mutations in breast cancer have been reported to date. There are also no reports of homozygous deletions or translocations affecting *BRCA1* in tumours. Evidence thus far, therefore, suggests that *BRCA1* may not be critical in the development of the majority of breast and ovarian cancers.

*Genetic heterogeneity*

In the BCLC study a clear distinction was apparent between breast-ovarian families and families containing breast cancer only (Easton *et al.*, 1993). All 57 families with at least one case of ovarian cancer were estimated to be linked, with a lower 95% confidence limit (CL) of 79% on the percentage linked. In contrast only an estimated 45% of families with multiple cases of breast cancer, but no ovarian cancer were linked (95% CL 25-66%). Linkage was more likely if the average age of breast cancer diagnosis in the family was young, although the distinction between the young and older age-at-onset families was not as striking as the age effect observed by Hall *et al.* (1990).

Since the original BCLC study, there have been a number of anecdotal reports of breast-ovarian families which are inconsistent with linkage to *BRCA1*. A pattern that has emerged however, is that it is those families with male breast cancer, some of which also contain ovarian cancer, which are not linked to *BRCA1*. A recent analysis of 132 breast-ovarian cancer families with no male breast cancer confirmed that the majority, although possibly not all, of these are due to *BRCA1* (Narod *et al.*, 1995). Overall an estimated 88% of families were linked (95% CL 74-97%) and in the subgroup of 81 families with at least two ovarian cancers 92% of families were linked (95% CL 76-100%). There are few families which contain multiple cases of ovarian cancer but no breast cancer, but all the evidence suggests that most if not all of these families are also caused by *BRCA1*. Steichen-Gersdorf *et al.* (1994) studied nine families with at least three cases of ovarian cancer and no cases of breast cancer diagnosed below age 50. Seven of the nine families were completely consistent with linkage to *BRCA1*.

In the original BCLC study only five families with any case of male breast cancer were included and evidence for or against linkage to *BRCA1* was inconclusive. In a more recent study of 22 families, each containing at least one male breast cancer case, strong evidence against linkage to *BRCA1* was demonstrated and the best estimate of the percentage of linked families was 0% with an upper 95% CL of 18% (Stratton *et al.*, 1994). Interestingly in the subgroup of 12 families containing male and female breast cancer and at least one ovarian cancer the evidence against linkage was also strong with an estimated 0% of families linked (95% CL 0-29%). Several of these families are now known to be due to *BRCA2* (see below).

*Associated breast and ovarian cancer risks*

In the future it should be possible to estimate directly the cancer risks conferred by *BRCA1* by studying gene carriers identified through large population-based studies. In the meantime, estimates have been derived from the data collected on linkage families using two essentially independent methods, namely maximising the LOD score over possible penetrance functions and using the incidence of second breast or ovarian cancers following a first breast cancer. Using either approach the overall lifetime risk of breast or ovarian cancer was estimated as close to 100% (Ford *et al.*, 1994; Easton *et al.*, 1995). Using the maximum LOD score method (assuming no heterogeneity of risk between families) the estimated cumulative risk for breast cancer rose to 51% by age 50 (95% CL 25-67%) and 85% by age 70 (95% CL 51-95%). The corresponding estimates for ovarian cancer were 23% by age 50 (95% CL 5-38%) and 63% by age 70 (95% CL 25-82%). Up to age 60 the second cancer data gave somewhat higher risks, possibly reflecting individual variation in risk due to other genetic or environmental factors or a tendency to ascertain families with individuals with double primary cancers, but neither the overall cumulative risk by age 70 nor the site-specific risks by age 70 differed between the studies. The largest *BRCA1* family to be reported in the literature, which contains some 30 breast cancers and 20 ovarian cancers, has also been analysed using lifetable analysis and provides further evidence for the high-lifetime risk estimates of breast and ovarian cancers (Goldgar *et al.*, 1994*b*). In this family the risks of breast and ovarian cancer by age 70 were 73% and 65% respectively (DE Goldgar and CM Lewis, personal communication).

As predicted by the segregation analyses, the age-specific incidence of breast cancer in *BRCA1* mutation carriers follows a markedly different pattern from that seen in the general population. The relative risk declines by an order of magnitude over the age range 30-70 years. The results for ovarian cancer are less clear, although there is some suggestion of a decline in relative risk with age (Easton *et al.*, 1995).

Simple inspection of the families which are likely to be linked to *BRCA1* suggests that the ovarian cancer risks are not homogeneous across families. Some of the large linked families contain only one or two cases of ovarian cancer, while other families contain more ovarian cancers than breast cancers. Easton *et al.* (1995) have shown that the observed patterns of disease are better explained by a model with two different susceptibility alleles (referred to as 'type 1' and 'type 2' alleles), one conferring a cumulative ovarian cancer risk of 26% by age 70 and the other conferring a cumulative ovarian cancer risk of 85%. There was no evidence to suggest that the breast cancer risk differed between families and under this model the estimated breast cancer risk by age 70 was 76%. The second allele, conferring a high ovarian cancer risk, was estimated to account for 11% of *BRCA1* mutations and the first allele (with a lower ovarian cancer risk), the remaining 89%. Some support for this allelic heterogeneity was found by considering the risks of ovarian cancer in individuals already affected with breast cancer, according to whether the family was probably due to the 'type 1' or 'type 2' allele. The risk of ovarian cancer in women with a previous breast cancer was higher in families with a posterior probability of greater than 50% of being due to a 'type 2' allele than in the remaining 'type 1' families, although only by a factor of two as compared with an estimated 6-fold difference in incidence rates in the heterogeneity analysis.

Now that *BRCA1* has been cloned it may prove possible to resolve the issue of heterogeneity by examining the disease phenotypes associated with different types of mutations, but it appears that large numbers of families will be required. In the collaborative survey of mutations in *BRCA1* reported by Shattuck-Eidens *et al.* (1995) there was not a statistically significant difference in the distribution of the *BRCA1* mutational spectra among low- and high-prevalence ovarian cancer families.

It is also conceivable that there are *BRCA1* mutations which confer a much lower overall risk of cancer than suggested here and do not usually generate the large multiple-case families suitable for linkage. If such mutations exist, then the estimates presented here would only be relevant to the 'high-risk' type families; however these are the families which are currently being counselled on the basis of linkage data in clinics.

It should be noted that all ovarian cancers due to *BRCA1* appear to be invasive epithelial tumours; there is no evidence that *BRCA1* can cause borderline or germ-cell tumours, or benign cysts. Equally, there is no evidence that *BRCA1* mutations (or, in fact, any of the other gene defects discussed here) result in lobular or ductal carcinoma *in situ* of the breast. There is some suggestion that *BRCA1*-linked ovarian cancers are more likely to be serous than mucinous (Narod, 1994).

*Increased risks of colon and prostate cancer*

Ford *et al.* (1994) studied 33 families with evidence for linkage to *BRCA1* to establish whether gene carriers are at an increased risk of any cancer other than breast or ovarian cancer. Eighty-seven other cancers were observed in individuals with breast or ovarian cancer and their first-degree relatives compared with 69.3 expected, based on national incidence rates. There were statistically significant excesses of colon cancer ($P = 0.002$) and prostate cancer ($P = 0.006$) but no significant excesses or deficits for any other sites. The maximum likelihood estimate of the relative risk of colon cancer in carriers compared with the general population was 4.11 (95% CL 2.36–7.15), giving an estimated cumulative risk of colon cancer in gene carriers of the order of 6% by age 70, compared with a 1–2% population risk. Although there was some suggestion of a higher risk in females compared with males this difference was non-significant. The maximum likelihood estimate of the relative risk of prostate cancer in carriers compared with the general population was 3.33 (95% CL 1.78–6.20). There was some suggestion that the relative risk of prostate cancer in *BRCA1* carriers is higher in the European families than in the American families, though this difference did not quite reach statistical significance (relative risks 8.49 and 2.48 respectively, $P = 0.06$). Since the incidence of prostate cancer is approximately 3-fold higher in the US than in Europe, the estimated absolute risk of prostate cancer is similar in the two populations and is approximately 8% by age 70. Such estimated absolute risks of colon and prostate cancer in carriers are probably too low to justify any particular preventive strategy; but this could alter if these cancers are found to be associated with particular *BRCA1* mutations.

### *The gene frequency of* BRCA1

In time it may be feasible to estimate the gene frequency of *BRCA1* by direct mutation testing of very large population-based series of samples. However, a reasonable estimate of the gene frequency has already been obtained indirectly by combining the penetrance estimates of *BRCA1* with the results of two population-based studies of cancer mortality in the relatives of breast and ovarian cancer patients (D Ford *et al.*, in preparation). In a study of cancer mortality in the first-degree relatives of 3295 breast cancer patients 49 ovarian cancer deaths below age 70 were observed compared with 33.71 expected at national rates, that is an excess of 15.29 deaths (Peto *et al.*, 1995). If *BRCA1* were responsible for the entire excess of ovarian cancer in relatives of breast cancer patients, then, given the penetrance estimates for *BRCA1*, the overall frequency of *BRCA1* would be 0.00064. The assumption that the familial association between breast and ovarian cancer is mainly due to *BRCA1* seems reasonable given the results of the BCLC study alluded to previously. Although *BRCA2* mutations may also cause ovarian cancer (see below) the risk is probably too low to have a measurable effect in population-based studies. In a companion study of cancer mortality in relatives of 1188 ovarian cancer patients 45 breast cancer deaths below age 70 were observed compared with 33.63 expected (Easton *et al.*, 1995). The same method applied to this study would predict a gene frequency for *BRCA1* of 0.00052. The best estimate of the gene frequency, taken as an average of the two estimates is 0.0006 (95% CL 0.0002–0.001). A gene frequency of 0.0006 corresponds to a carrier frequency of 1 in 800. Even taking the upper 95% CL only 1 in 500 women carries the mutation. Table II shows the proportions of breast and ovarian cancers which would be attributable to germline high penetrance *BRCA1* mutations in each age group for possible gene frequencies, 0.0002, 0.0006 and 0.001. Much higher frequencies of the order of 1 in 300 are often cited, but these are derived from segregation analyses of breast cancer (e.g. Claus *et al.*, 1991) and do not relate to *BRCA1* specifically.

### The *BRCA2* gene

### *Localisation of* BRCA2

Wooster *et al.* (1994) have recently localised a second breast cancer susceptibility gene (*BRCA2*) to 13q12–13. They typed 15 families with multiple cases of early-onset breast cancer and evidence against linkage to *BRCA1*. A multipoint LOD score of 11.65 was obtained using markers D13S260 and D13S267 with an estimated 74% (95% CL 35–97%) of families linked. A map of the region is shown in Figure 1. Single recombinants in breast cancer cases aged 43 and 39 in two large families with convincing evidence for linkage place *BRCA2* telomeric to D13S289. One recombinant, also in a large family, in a bilateral breast cancer diagnosed at ages 38 and 41 places the gene centromeric to D13S267.

Although the tumour-suppressor gene *RB1* is in the same region it is already clear that *BRCA2* is not *RB1* since there are a number of recombinants between *RB1* and the disease in linked families. Other candidate genes within 13q11–14 include members of a family of tyrosine kinase genes that are related to the *FMS* protooncogene (Rosnet *et al.*, 1993) and the *FTE1* gene, which may act as an effector of the v-*fos* oncogene and is a mammalian homologue of a yeast gene

**Figure 1** Genetic map of chromosome 13q in the region of *BRCA2*. Genetic distances between markers are given in centimorgans (cM).

**Table II** Estimated percentage of cases due to germline *BRCA1* mutations

| | *Gene frequency 0.0002* | | *Gene frequency 0.0006* | | *Gene frequency 0.001* | |
|---|---|---|---|---|---|---|
| *Age group* | *Breast* | *Ovary* | *Breast* | *Ovary* | *Breast* | *Ovary* |
| 20–29 | 2.6 | 2.0 | 7.5 | 5.9 | 11.9 | 9.5 |
| 30–39 | 1.7 | 1.9 | 5.1 | 5.6 | 8.2 | 9.0 |
| 40–49 | 0.7 | 1.6 | 2.2 | 4.6 | 3.6 | 7.4 |
| 50–59 | 0.5 | 0.9 | 1.4 | 2.6 | 2.4 | 4.3 |
| 60–69 | 0.3 | 0.6 | 0.8 | 1.8 | 1.3 | 2.9 |
| 20–69 | 0.6 | 1.0 | 1.7 | 2.8 | 2.8 | 4.6 |

involved in protein import into mitochondria (Kho and Zarbl, 1992). Preliminary evidence suggests that *BRCA2* acts as a tumour-suppressor gene. Collins *et al.* (1995) have demonstrated loss of heterozygosity in seven out of eight breast cancers from one large linked family, with all losses involving the wild-type chromosome.

*Associated cancer risks*

Like *BRCA1*, *BRCA2* appears to confer a high risk of early-onset breast cancer in females; in a previous segregation analysis of the largest *BRCA2*-linked family (Utah 107) the risk by age 70 was estimated as 63%, rising to 87% by age 80 (Bishop *et al.*, 1988). However, the ovarian cancer risk conferred by *BRCA2* is likely to be much lower than the risk conferred by *BRCA1*; in the two families showing the strongest evidence of linkage to *BRCA2* (multipoint LOD score greater than 3.0) there are 49 reported cases of breast cancer, including 39 under 50, and only three ovarian cancers (excluding affecteds who do not carry the linked haplotype). In contrast it seems likely that the male breast cancer risk in carriers of *BRCA2* mutations, although small, is probably greater than in male *BRCA1* mutation carriers. In the same two families there were four cases of male breast cancer and in a further three families which showed evidence of linkage to *BRCA2* there were single cases of affected males.

Collaborative studies will now be undertaken by the BCLC to define more precisely the proportions of families of different types linked to *BRCA2*, to estimate the penetrance of the *BRCA2* gene and to investigate the possibility that mutations in *BRCA2* cause other cancers.

### Hereditary non-polyposis colorectal cancer genes

Hereditary non-polyposis colorectal cancer (HNPCC) is a dominant familial cancer syndrome which predisposes to colon cancer, and to a lesser extent, cancers at other sites including endometrium, stomach, small bowel and ovary. Watson and Lynch (1993) estimated the risk of ovarian cancer in members of 23 HNPCC kindreds to be approximately 4-fold greater than in the general population. The true relative risk will be slightly higher since not all those individuals studied will be carriers of the deleterious gene. The median age of diagnosis was 40 years, which is considerably lower than the median age of diagnosis in the general population and in interesting contrast to ovarian cancers within *BRCA1*-linked families. There was some evidence that the ovarian cancer risk differed between families.

It is now known that HNPCC can result from germline mutations in a class of DNA repair genes originally identified in yeast. To date, four such genes are known to cause HNPCC, namely the *hMSH2*, *hMLH1*, *hPMS1* and *hPMS2* genes on chromosomes 2p, 3p and 7p (Leach *et al.*, 1993; Bronner *et al.*, 1994; Nicolaides *et al.*, 1994; Papadopoulos *et al.*, 1994). However it is unclear whether all of these genes confer an increased risk of ovarian cancer.

The frequency of these HNPCC mutations is the subject of some debate. Some reports have suggested that as much as 20% of colorectal cancer could be due to mutations in these genes. These estimates are based on the proportions of colorectal tumours exhibiting the microsatellite instability phenotype, which is common in HNPCC families. However, most of these tumours are likely to be sporadic and as yet there is no evidence that this phenotype is invariably the result of germline mutations in mismatch repair genes. Clinical studies suggest that the HNPCC syndrome probably only accounts for 1% or less of colorectal cancer (Bodmer *et al.*, 1994; Aaltonen *et al.*, 1994), suggesting a population frequency of the order of one in 10 000.

### The Li–Fraumeni syndrome and the *TP53* gene

The Li–Fraumeni familial cancer syndrome (LFS) is a rare dominant syndrome characterised by young onset sarcomas, premenopausal breast cancers, brain tumours, adrenocortical tumours and other cancers (Li *et al.*, 1988). Germline mutations in the *TP53* gene have been identified in over 50% of LFS families and in a number of families with some but not all of the features of the classical LFS (Malkin *et al.*, 1990; Eeles, 1993).

The absolute risk of breast cancer in mutation carriers has not been estimated precisely but would appear to be at least 50% by age 50, with little increase in risk after menopause (Li *et al.*, 1988). Available evidence suggests that germline *TP53* mutations account for a very small proportion of breast cancer outside the LFS (or Li–Fraumeni-like syndrome). Although a splice-site *TP53* mutation has been found in one predominantly breast–ovarian cancer family including two breast cancer cases diagnosed before age 35, an ovarian cancer at 31 and a childhood choroid plexus tumour (Jolly *et al.*, 1994), it is now clear that most breast–ovarian families are caused by *BRCA1* and some, possibly all, of the remainder are due to *BRCA2*. In population based studies of breast cancer cases *TP53* germline mutations have been found in less than 1% of cases, even at young ages (Borresen *et al.*, 1992; Sidransky *et al.*, 1992).

### Other breast cancer genes with high penetrance

There are a number of families characterised by early-onset female breast cancer and/or male breast cancer which show evidence against linkage to both *BRCA1* and *BRCA2* (Wooster *et al.*, 1994; MR Stratton, R Wooster and DE Goldgar, personal communication). It seems possible, therefore, that at least one more breast cancer gene with high penetrance remains to be discovered. It should be noted, however, that some of these apparently unlinked families will be due to *BRCA1* or *BRCA2*, but appear unlinked because of young sporadic cases occurring by chance. Indeed *BRCA1* mutations have been found in a number of such families (DE Goldgar, personal communication). A formal study to estimate the proportion of high-risk families not accounted for by *BRCA1* and *BRCA2* is currently being conducted by the BCLC.

### The ataxia-telangiectasia gene

Ataxia-telangiectasia (AT) is an autosomal recessive disorder characterised by cerebellar ataxia, oculocutaneous telangiectasia, a hypersensitivity to ionising radiation and an increased susceptibility to cancer (McKinnon, 1987; Sedgwick and Boder, 1991). The frequency of the AT gene, which is located on chromosome 11q22–23, is likely to be of the order of 0.005 (Easton, 1994), suggesting that about 1% of the population are heterozygous for AT.

A number of studies have shown that female relatives of AT patients are at an increased risk of breast cancer. Easton (1994), in a review of four published studies (Swift *et al.*, 1987; Pippard *et al.*, 1988; Borresen *et al.*, 1990; Swift *et al.*, 1991), estimated that the relative risk of breast cancer to AT heterozygotes is 3.9 fold (95% CL 2.1–7.2). A gene conferring such a low relative risk would not, on the whole give rise to multiple early-onset case families. For example, assuming a relative risk of 3.9 and a gene frequency of 0.005 Easton (1994) estimated that only 1% of affected sister pairs aged 35 at diagnosis would be due to AT, the percentage rising to 3% of sister pairs diagnosed at age 55. (This assumes a familial relative risk of breast cancer of 5.3 at age 35, falling to 1.5 at age 55, based on Claus *et al.*, 1990*b*). The proportion of families with three or more affected first-degree relatives which could be attributed to AT would be even lower.

Assuming a constant relative risk of 3.9 across all ages and a gene frequency of 0.005, the AT gene would account for about 4% of breast cancers diagnosed below age 60 (95% CL 1–13%). However, there is some suggestion that the relative risk declines with age (Easton, 1994). If the AT gene confers a relative risk of breast cancer of 9 below age 40, 3

between 40 and 60 and 1 above age 60, AT could be responsible for 8% of cases under 40 and 2% of cases between 40 and 60.

### *HRAS1*

The proto-oncogene *HRAS1* is tightly linked to a minisatellite locus, which has some 30 alleles of which four represent 94% of all alleles in whites, the remainder being rare (Capon *et al.*, 1983; Krontiris *et al.*, 1986; Krontiris, 1990). A meta-analysis of 23 studies has shown a highly significant association of rare *HRAS1* alleles with cancer (Krontiris *et al.*, 1993). It is undetermined as yet whether the *HRAS1* gene itself or a closely linked gene is responsible for the association; if it is due to the *HRAS1* gene, the mechanism underlying the association is completely unclear. Sites for which significant associations were found were breast, colorectum, urinary bladder and acute leukaemia. The relative risk of breast cancer associated with rare *HRAS1* alleles was 1.68 (95% CL 1.23–2.29), and the authors estimated that as many as 1 in 11 breast cancers might be attributed to this factor.

### Conclusions

Studies of multiple-case families have led to the identification of highly penetrant genes which predispose to breast and ovarian cancer. *BRCA1* and *BRCA2* appear to account for the majority of large multiple-case families, but it is less clear to what extent they account for the more common, smaller breast and ovarian cancer families such as pairs of affected relatives, since these could be due to lower penetrance genes. For *BRCA1* at least, it should now be possible to answer this question directly.

From a genetic counselling standpoint it is already possible to identify women in families linked to *BRCA1* and to counsel them on the basis of linkage data and reasonably precise estimates of cancer risk. Now that *BRCA1* has been cloned it may not be long before direct testing for *BRCA1* mutations becomes practical on a routine basis in high-risk women. However, it is already clear that mutations will be widely scattered throughout the gene, thus testing on a large scale is likely to be laborious and it will be difficult to exclude the presence of a mutation. This, combined with the rarity of *BRCA1* mutations makes it unlikely that testing in the general population will be useful, other than for research purposes. Intense efforts will now be made to clone *BRCA2*. If *BRCA2* is homologous to *BRCA1* or is one of the candidate genes already identified, *BRCA2* may be cloned very quickly, otherwise the time scale involved is indeterminate. In the meantime risk estimates for *BRCA2* should be available soon, allowing women in *BRCA2*-linked families to have counselling on the basis of genotyping.

Epidemiological studies are currently being conducted by the BCLC to assess whether or not the risks of breast and ovarian cancer in *BRCA1* carriers are substantially modified by other risk factors such as parity, age at first birth and oral contraceptive use. From a practical point of view the most important risk factor is oral contraceptive use. In the general population long-term oral contraceptive use is known to reduce substantially the risk of ovarian cancer (Parazzinni *et al.*, 1991) but to increase slightly the risk of breast cancer at young ages (UK National Case–Control Study Group, 1990), so their use in *BRCA1* carriers might cause a marked increase or decrease in overall cancer risk. Similar studies of *BRCA2* carriers will also be important.

Taken together the high penetrance genes (*BRCA1*, *BRCA2*, *TP53* and the HNPCC genes) are likely to account for considerably less than 10% of all breast and ovarian cancer cases in the population. Common susceptibility genes could account for a much higher proportion of cancers in the general population but would necessarily confer much lower lifetime risks. The *HRAS1* minisatellite locus is an example of a common, low penetrant gene which causes breast cancer but others may exist. The identification and understanding of such genes (if they exist) would be of great practical importance, possibly leading to genetic testing as an adjunct to screening for common cancers. However, low penetrant genes will not cause multiple case families and may not even give rise to a detectably increased risk in relatives of cancer patients; thus they are likely to be difficult to detect by linkage and will only be identified through associations of cancer risk with other heritable phenotypes (as in the case of AT), or through direct testing of candidate genes.

**Acknowledgements**
The Institute of Cancer Research receives financial support from the Cancer Research Campaign and the Medical Research Council.

### References

AALTONEN LA, SANKILA R, MECKLIN J-P, JARVINEN H, PUKKALA E, PELTOMAKI P AND DELACHAPELLE A. (1994). A novel approach to estimate the proportion of hereditary nonpolyposis colorectal cancer of total colorectal cancer burden. *Cancer Det. Prev.*, **18**, 57–63.

BISHOP DT, CANNON-ALBRIGHT L, McLELLAN T, GARDNER EJ AND SKOLNICK MH. (1988). Segregation and linkage analysis of 9 Utah breast cancer pedigrees. *Genet. Epidemiol.*, **5**, 151–169.

BODMER W, BISHOP T AND KARRAN P. (1994). Genetic steps in colorectal cancer. *Nat. Genet.*, **6**, 217–219.

BORRESEN A-L, ANDERSEN TI, TRETI S, HEIBERG A AND MOLLER P. (1990). Breast cancer and other cancers in Norwegian families with ataxia-telangiectasia. *Genes Chrom. Cancer*, **2**, 339–340.

BORRESEN A-L, ANDERSEN TI, GARBER J, BARBIERPIRAUX N, THORLACIUS S, EYFJORD J, OTTESTAD L, SMITHSORENSEN B, HOVIG E, MALKIN D AND FRIEND SH. (1992). Screening for germ line TP53 mutations in breast cancer patients. *Cancer Res.*, **52**, 3234–3236.

BROCCA PP. (1866). *Traites des tumeurs*, **1**, 80.

BRONNER CE, BAKER SM, MORRISON PT, WARREN G, SMITH LG, LESCOE MK, KANE M, EARABINO C, LIPFORD J, LINDBLOM A, TANNERGARD P, BOLLAG RJ, GODWIN AR, WARD DC, NORDENSKJOLD M, FISHEL R, KOLODNER R AND LISKAY RM. (1994). Mutation in the DNA mismatch repair gene homologue hMLH1 is associated with hereditary nonpolyposis colon cancer linked to chromosome 3p. *Nature*, **368**, 258–261.

CAPON DJ, CHEN EY, LEVINSON AD, SEEBURG PH AND GOEDDEL DV. (1983). Complete nucleotide sequences of the T24 human bladder carcinoma oncogene and its normal homologue. *Nature*, **302**, 33–37.

CLAUS EB, RISCH N AND THOMPSON WD. (1990*a*). Age of onset as an indicator of familial risk of breast cancer. *Am. J. Epidemiol.*, **131**, 961–972.

CLAUS EB, RISCH N AND THOMPSON WD. (1990*b*). Using age of onset to distinguish between subforms of breast cancer. *Ann. Hum. Genet.*, **54**, 169–177.

CLAUS EB, RISCH N AND THOMPSON WD. (1991). Genetic analysis of breast cancer in the cancer and steroid hormone study. *Am. J. Hum. Genet.*, **48**, 232–242.

COLLINS N, MCMANUS R, WOOSTER R, MANGION J, SEAL S, SUNIL L, ORMISTON W, DALY P, FORD D, EASTON DF AND STRATTON MR. (1995). Consistent loss of the wild type allele in breast cancers from a family linked to the BRCA2 gene on chromosome 13q12-13. *Oncogene*, **10**, 1673–1675.

CORNELIS RS, DEVILEE P, VAN VLIET M, KUIPERSDIJ K, SHOORN N, KERSENMAEKER A, BARDOEL A, KHAN PM AND CORNELISSE CJ. (1993). Allele loss patterns on chromosome 17q in 109 breast carcinomas indicate at least two distinct target regions. *Oncogene*, **8**, 781–785.

EASTON DF. (1994). Cancer risks in A-T heterozygotes. *Int. J. Radiat. Biol.*, **66**, S177–182.

EASTON DF, BISHOP DT, FORD D AND CROCKFORD GP., THE BREAST CANCER LINKAGE CONSORTIUM. (1993). Genetic linkage analysis in familial breast and ovarian cancer: results from 214 families. *Am. J. Hum. Genet.*, **52**, 678-701.

EASTON DF, FORD D AND BISHOP DT., BREAST CANCER LINKAGE CONSORTIUM. (1995). Breast and ovarian cancer incidence in BRCA1 mutation carriers. *Am. J. Hum. Genet.*, **56**, 265-271.

EASTON DF, MATTHEWS FE, FORD D, SWERDLOW AJ AND PETO J. (1995). Cancer risks to relatives of ovarian cancer patients. *Int. J. Cancer*, (in press).

EELES RA. (1993). Predictive testing for germline mutations in the p53 gene: are all the questions answered? *Eur. J. Cancer*, **29a (10)**, 1361-1365.

FORD D, EASTON DF, BISHOP DT, NAROD SA AND GOLDGAR DE. BREAST CANCER LINKAGE CONSORTIUM. (1994). Risks of cancer in BRCA1 mutation carriers. *Lancet*, **343**, 692-695.

FORD D, EASTON DF AND PETO J. Estimates of the gene frequency of BRCA1 and its contribution to breast and ovarian cancer incidence. (in preparation).

FUTREAL PA, LIU Q, SHATTUCK-EIDENS D, COCHRAN C, HARSHMAN K, TAVTIGIAN S, BENNETT LM, HAUGENSTRANO A, SWENSEN J, MIKI Y, EDDINGTON K, McCLURE M, FRYE C, WEAVERFELDHAUS J, DING W, GHOLAMI Z, SODERKVIST P, TERRY L, JHANWAR S, BERCHUCK A, IGLEHART JD, MARKS J, BALLINGER DG, BARRETT JC, SKOLNICK MH, KAMB A AND WISEMAN R. (1994). BRCA1 mutations in primary breast and ovarian carcinomas. *Science*, **266**, 120-122.

GATTI RA, BERKEL I, BODER E, BRAEDT G, CHARMLEY P, CONCANNON P, ERSOY F, FOROUD T, JASPERS NGJ, LANGE K, LATHROP GM, LEPPERT M, NAKAMURA Y, O'CONNELL P, PATERSON M, SALSER W, SANAL O, SILVER J, SPARKES RS, SUSI E, WEEKS DE, WEI S, WHITE R AND YODER F. (1988). Localisation of an ataxia-telangiectasia gene to chromosome 11q22-23. *Nature*, **336**, 577-580.

GOLDGAR DE, EASTON DF, CANNON-ALBRIGHT LA AND SKOLNICK MH. (1994*a*). A systematic population based-assessment of cancer risk in first degree relatives of cancer probands. *J. Natl. Cancer Inst.*, **86**, 1600-1608.

GOLDGAR DE, FIELDS P, LEWIS CM, TRAN TD, CANNON-ALBRIGHT LA, WARD JH, SWENSEN J AND SKOLNICK MH. (1994*b*). A large kindred with 17q-linked breast and ovarian cancer - genetic, phenotypic and genealogical analysis. *J. Natl. Cancer Inst.*, **86**, 200-209.

GRODEN J, THLIVERIS A, SAMOWITZ W, CARLSON M, GELBERT L, ALBERTSEN H, JOSLYN G, STEVENS J, SPIRIO L, ROBERTSON M, SARGEANT L, KRAPCHO K, WOLFF E, BURT R, HUGHES JP, WARRINGTON J, McPHERSON J, WASMUTH J, LEPASLIER D, ABDERRAHIM H, COHEN D, LEPPERT M AND WHITE R. (1991). Identification and characterization of the familial adenomatous polyposis coli gene. *Cell*, **66**, 589-600.

HALL JM, LEE MK, NEWMAN B, MORROW JE, ANDERSON LA, HUEY B AND KING M-C. (1990). Linkage of early onset familial breast cancer to chromosome 17q21. *Science*, **250**, 1684-1689.

HOULSTON RS, COLLINS A, SLACK J, CAMPBELL S, COLLINS WP, WHITEHEAD MI AND MORTON NE. (1991). Genetic epidemiology of ovarian cancer: segregation analysis. *Ann. Hum. Genet.*, **55**, 291-299.

HOULSTON RS AND PETO J. (1995). Genetics and the common cancers. In: *Genetic Predisposition to Cancer*, R Eeles, B Ponder, D Easton, A Horwich (Eds). Chapman and Hall: London.

HUANG H-JS, YEE JK, SHEW JY, CHEN PL, BOOKSTEIN R, FRIEDMANN T, LEE EYHP AND LEE WH. (1988). Suppression of the neoplastic phenotype by replacement of the RB gene in human cancer cells. *Science*, **242**, 1563-1566.

JACOBS IJ, SMITH SA, WISEMAN RW, FUTREAL PA, HARRINGTON T, OSBORNE RT, LEECH V, MOLYNEUX A, BERCHUCK A, PONDER BAJ AND BAST RC. (1993). A deletion unit on chromosome 17q in epithelial ovarian tumors distal to the familial breast/ovarian cancer locus. *Cancer Res.*, **53**, 1218-1221.

JOLLY KW, MALKIN D, DOUGLAS EC, BROWN TF, SINCLAIR AE AND LOOK AT. (1994). Splice-site mutation of the p53 gene in a family with hereditary breast-ovarian cancer. *Oncogene*, **9**, 97-102.

KELSELL DP, BLACK DM, BISHOP DT AND SPURR NK. (1993). Genetic analysis of the BRCA1 region in a large breast/ovarian family: refinement of the minimal region containing BRCA1. *Hum. Mol. Genet.*, **2**, 1823-1828.

KHO CJ AND ZARBL H. (1992). FTE1, A V-FOS transformation effector gene, encodes the mammalian homolog of a yeast gene involved in protein import into mitochondria. *Proc. Natl Acad. Sci. USA.*, **89**, 2200-2204.

KINZLER KW, NILBERT MC, SU LK, VOGELSTEIN B, BRYAN TM, LEVY DB, SMITH KJ, PREISINGER AC, HEDGE P, McKECHNIE D, FINNIEAR R, MARKHAM A, GROFFEN J, BOGUSKI MS, ALTSCHUL SF, HORII A, ANDO H, MIYOSHI Y, MIKI Y, NISHISHOI I AND NAKAMURA Y. (1991). Identification of FAP locus genes from chromosome 5q21. *Science*, **253**, 661-665.

KNUDSON AG. Jr. (1971). Mutation and cancer: statistical study of retinoblastoma. *Proc. Natl Acad. Sci. USA*, **68**, 820-823.

KRONTIRIS TG, DiMARTINO NA, COLB M, MITCHESON HD AND PARKINSON DR. (1986). Human restriction fragment length polymorphisms and cancer risk assessment. *J. Cell. Biochem.*, **30**, 319-329.

KRONTIRIS TG. (1990). Detection of cancer predisposition by hypervariable region analysis. In: *Detection of Cancer Predisposition: Laboratory Approaches*, L Spatz, AD Bloom, NW Paul (Eds). pp. 129-140. March of Dimes Birth Defects Foundation: White Plains, NY.

KRONTIRIS TG, DEVLIN B, KARP DD, ROBERT NJ AND RISCH N. (1993). An association between the risk of cancer mutations in the HRAS1 minisatellite locus. *N. Engl. J. Med.*, **329**, 517-523.

LEACH FS, NICOLAIDES NC, PAPADOPOULOS N, LIU B, JEN J, PARSONS R, PELTOMAKI P, SISTONEN P, AALTONEN LA, NYSTROMLAHTI M, GUAN XY, ZHANG J, MELTZER PS, YU JW, KAO FT, CHEN DJ, CEROSALETTI KM, FOURNIER REK, TODD S, LEWIS T, LEACH RJ, NAYLOR SL, WEISSENBACH J, MECKLIN JP, JARVINEN H, PETERSEN GM, HAMILTON SR, GREEN J, JASS J, WATSON P, LYNCH HT, TRENT JM, DELACHAPELLE A, KINZLER KW AND VOGELSTEIN B. (1993). Mutations of a mutS homolog in hereditary nonpolyposis colorectal cancer. *Cell*, **75**, 1215-1225.

LI FP, FRAUMENI JF, Jr. MULVIHILL JJ, BLATTNER WA, DREYFUS MG, TUCKER MA AND MILLER RW. (1988). A cancer family syndrome in twenty-four kindreds. *Cancer Res.*, **48**, 5358-5362.

LOBACCARO J-M, LUMBROSO S, BELON C, GALTIERDEREURE F, BRINGER J, LESIMPLE T, NAMER M, CUTULI BF, PUJOL H AND SULTAN C. (1993). Androgen receptor gene mutation in male breast cancer. *Hum. Mol. Genet.*, **2**, 1799-1802.

LYNCH HT, KRUSH AJ, LEMON HM, KAPLAN AR, CANDIT PT AND BOTTOMLEY RH. (1972). Tumour variations in families with breast cancer. *JAMA*, **220**, 1631-1635.

McKINNON PJ. (1987). Ataxia-telangiectasia: an inherited disorder of ionizing-radiation sensitivity in man. Progress in the elucidation of the underlying biochemical defect. *Hum. Genet.*, **75**, 197-208.

MALKIN D, LI FP, STRONG LC, FRAUMENI JF, NELSON CE, KIM DH, KASSEL J, GRYKA MA, BISCHOFF FZ, TAINSKY MA AND FRIEND SH. (1990). Germline p53 mutations in a familial syndrome of breast cancer, sarcomas and other neoplasms. *Science*, **250**, 1233-1238.

MERAJVER SD, PHAM TM, CADUFF RF, CHEN M, POY EL, COONEY KA, WEBER BL, COLLINS FS, JOHNSTON C AND FRANK TS. (1995). Somatic mutations in the BRCA1 gene in sporadic ovarian tumours. *Nat. Genet.* **9**, 439-443.

MIKI Y, SWENSEN J, SHATTUCK-EIDENS D, FUTREAL PA, HARSHMAN K, TAVTIGIAN S, LIU QY, COCHRAN C, BENNETT LM, DING W, BELL R, ROSENTHAL J, HUSSEY C, TRAN T, McCLURE M, FRYE C, HATTIER T, PHELPS R, HAUGENSTRANO A, KATCHER H, YAKUMO K, GHOLAMI Z, SHAFFER D, STONE S, BAYER S, WRAY C, BOGDEN R, DAYANANTH P, WARD J, TONIN P, NAROD S, BRISTOW PK, NORRIS FH, HELVERING L, MORRISON P, ROSTECK P, LAI M, BARRETT JC, LEWIS C, NEUHAUSEN S, CANNON-ALBRIGHT L, GOLDGAR D, WISEMAN R, KAMB A AND SKOLNICK MH. (1994). Isolation of BRCA1, the 17q-linked breast and ovarian cancer susceptibility gene. *Science*, **266**, 66-71.

NAROD SA. (1994). Genetics of breast and ovarian cancer. *Br. Med. Bull.*, **50**, 656-676.

NAROD SA, FEUNTEUN J, LYNCH HT, WATSON P, CONWAY T, LYNCH J AND LENOIR GM. (1991). Familial breast-ovarian cancer locus on chromosome 17q12-23. *Lancet*, **338**, 82-83.

NAROD SA, FORD D, DEVILEE P, BARKARDOTTIR RB, LYNCH HT, SMITH SA, PONDER BAJ, WEBER BL, GARBER JE, BIRCH JM, CORNELIS RS, KELSELL DP, SPURR NK, SMYTH E, HAITES N, SOBOL H, BIGNON YJ, CHANG-CLAUDE J, HAMANN U, LINDBLOM A, BORG A, PIVER MS, GALLION HH, STRUEWING JP, WHITTEMORE A, TONIN P, GOLDGAR DE AND EASTON DF, THE BREAST CANCER LINKAGE CONSORTIUM. (1995). An evaluation of genetic heterogeneity in 145 breast-ovarian cancer families. *Am. J. Hum. Genet.*, **56**, 254-264.

NICOLAIDES NC, PAPADOPOULOS N, LIU B, WEI YF, CARTER KC, RUBEN SM, ROSEN CA, HASELTINE WA, FLEISCHMANN RD, FRASER CM, ADAMS MD, VENTER JC, DUNLOP MG, HAMILTON SR, PETERSEN GM, DELACHAPELLE A, VOGELSTEIN B AND KINZLER KW. (1994). Mutations of two PMS homologues in hereditary nonpolyposis colon cancer. *Nature*, **371**, 75-80.

OLSHAUSEN. (1877). Die Krankheiten der Ovarian. F. Enke: Stuttgart.

PAPADOPOULOS N, NICOLAIDES NC, WEI YF, RUBEN SM, CARTER KC, ROSEN CA, HASELTINE WA, FLEISCHMANN RD, FRASER CM, ADAMS MD, VENTER JC, HAMILTON SR, PETERSEN GM, WATSON P, LYNCH HT, PELTOMAKI P, MECKLIN JP, DELACHAPELLE A, KINZLER KW AND VOGELSTEIN B. (1994). Mutation of the mutL homolog associated with hereditary colon cancer. *Science*, **263**, 1625-1629.

PARAZZINNI F, FRANCHESI S, LA VECCHIA C AND FASOLI M. (1991). The epidemiology of ovarian cancer. *Gynecol. Oncol.*, **43**, 9-23.

PETO J, EASTON DF, MATTHEWS FE, FORD D AND SWERDLOW AJ. (1995). Cancer mortality in relatives of breast cancer patients. *Int. J. Cancer*, (in press).

PIPPARD EC, HALL AJ, BARKER DJP AND BRIDGES BA. (1988). Cancer in homozygotes and heterozygotes of ataxia-telangiectasia and xeroderma pigmentosum in Britain. *Cancer Res.*, **48**, 2929-2932.

ROSNET O, STEPHENSON D, MATTEI MG, MARCHETTO S, SHIBUYA M, CHAPMAN VM AND BIRNBAUM D. (1993). Close physical linkage of the FLT1 and FLT3 genes on chromosome 13 in man and chromosome 5 in mouse. *Oncogene*, **8**, 173-179.

SCHILDKRAUT JM, RISCH N AND THOMPSON WD. (1989). Evaluating genetic association among ovarian, breast, and endometrial cancer: evidence for a breast/ovarian cancer relationship. *Am. J. Hum. Genet.*, **45**, 521-529.

SEDGWICK RP AND BODER E. (1991). Ataxia-telangiectasia. In: *Hereditary Neuropathies and Spinocerebellar Atrophies, Handbook of Clinical Neurology*, Vol 16. PJ Vinken, GW Bruyn, HL Klawans (Eds). pp. 347-423. Elsevier: Amsterdam.

SHATTUCK-EIDENS D, MCCLURE M, SIMARD J, LABRIE F, NAROD S, COUCH F, HOSKINS K, WEBER B, CASTILLA L, ERDOS M, BRODY L, FRIEDMAN L, OSTERMEYER E, SZABO C, KING M-C, JHANWAR S, OFFIT K, NORTON L, GILEWSKI T, LUBIN M, OSBORNE M, BLACK D, BOYD M, STEEL M, INGLES S, HAILE R, LINDBLOM A, OLSSON H, BORG A, BISHOP DT, SOLOMON E, RADICE P, SPATTI G, GAYTHER S, PONDER B, WARREN W, STRATTON M. LIU Q, FUJIMURA F, LEWIS C, SKOLNICK MH AND GOLDGAR DE. (1985). A collaborative survey of 80 mutations in the BRCA1 breast and ovarian cancer susceptibility gene. *JAMA*, 535-541.

SIDRANSKY D, TOKINO T, HELZLSOUER K, ZEHNBAUER B, RAUSCH G, SHELTON B, PRESTIGIACOMO L, VOGELSTEIN B AND DAVIDSON N. (1992). Inherited p53 gene mutations in breast cancer. *Cancer Res.*, **52**, 2984-2986.

SMITH SA, EASTON DF, EVANS DGR AND PONDER BAJ. (1992). Allele losses in the region 17q12-21 in familial breast and ovarian cancer involve the wild-type chromosome. *Nat. Genet.*, **2**, 128-131.

STEICHEN-GERSDORF E, GALLION HH, FORD D, GIRODET C, EASTON DF, DiCIOCCIO RA, EVANS G, PONDER MA, PYE C, MAZOYER S, NOGUCHI T, KARENGUEVEN F, SOBOL H, HARDOUIN A, BIGNON YJ, PIVER MS, SMITH SA AND PONDER BAJ. (1994). Familial site-specific ovarian cancer is linked to BRCA1 on 17q12-21. *Am. J. Hum. Genet.*, **55**, 870-875.

STRATTON MR, FORD D, NEUHASEN S, SEAL S, WOOSTER R, FRIEDMAN LS, KING M-C, EGILSSON V, DEVILEE P, MCMANUS R, DALY PA, SMYTH E, PONDER BAJ, PETO J, CANNON-ALBRIGHT L, EASTON DF AND GOLDGAR DE. (1994). Familial male breast cancer is not linked to BRCA1. *Nat. Genet.*, **7**, 103-107.

SWIFT M, REITNAUER PJ, MORRELL D AND CHASE CL. (1987). Breast and other cancers in families with ataxia-telangiectasia. *N. Engl. J. Med.*, **316**, 1289-1294.

SWIFT M, MORRELL D, MASSEY RB AND CHASE CL. (1991). Incidence of cancer in 161 families affected with ataxia-telangiectasia. *N. Engl. J. Med.*, **325**, 1831-1836.

UK NATIONAL CASE-CONTROL STUDY GROUP. (1990). Oral contraceptive use and breast cancer risk in young women: subgroup analysis. *Lancet*, **335**, 1507-1509.

WATSON P AND LYNCH HT. (1993). Extracolonic cancer in hereditary nonpolyposis colorectal cancer. *Cancer*, **71**, 677-685.

WOOSTER R, MANGION J, EELES R, SMITH S, DOWSETT M, AVERILL D, BARRETTLEE P, EASTON DF, PONDER BAJ AND STRATTON MR. (1992). A germline mutation in the androgen receptor in two brothers with breast cancer and Reifenstein syndrome. *Nat. Genet.*, **2**, 132-134.

WOOSTER R, NEUHAUSEN S, MANIGION J, QUIRK Y, FORD D, COLLINS N, NGUYEN K, SEAL S, TRAN T, AVERILL D, FIELDS P, MARSHALL G, NAROD S, LENOIR GM, LYNCH H, FEUNTEUN J, DEVILEE P, CORNELISSE CJ, MENKO FH, DALY PA, ORMISTON W, MCMANUS R, PYE C, LEWIS CM, CANNON-ALBRIGHT LA, PETO J, PONDER BAJ, SKOLNICK MH, EASTON DF, GOLDGAR DE AND STRATTON MR. (1994). Localisation of a breast cancer susceptibility gene (BRCA2) to chromosome 13q by genetic linkage analysis. *Science*, **265**, 2088-2090.